# Supplementary material for: Conceptualisation and psychometric evaluation of positive psychological outcome measures used in adolescents and young adults living with HIV: a mixed scoping and systematic review protocol
Source: BMJ Open. 2022 Sep 21;12(9):e066129. doi: 10.1136/bmjopen-2022-066129 (PMC9494580; doi:10.1136/bmjopen-2022-066129)
Supplement: Author's manuscript [file bmjopen-2022-066129.draft_revisions.pdf]

BMJ Open is committed to open peer review. As part of this commitment we make the peer review history of every article we publish publicly available.

When an article is published we post the peer reviewers' comments and the authors' responses online. We also post the versions of the paper that were used during peer review. These are the versions that the peer review comments apply to.

The versions of the paper that follow are the versions that were submitted during the peer review process. They are not the versions of record or the final published versions. They should not be cited or distributed as the published version of this manuscript.

BMJ Open is an open access journal and the full, final, typeset and author-corrected version of record of the manuscript is available on our site with no access controls, subscription charges or pay-per-view fees (http://bmjopen.bmj.com).

If you have any questions on BMJ Open's open peer review process please email info.bmjopen@bmj.com

A conceptualisation and psychometric evaluation of positive psychological outcome measures used in adolescents and young adults living with HIV: a mixed scoping and systematic review protocol.

Journal:	*BMJ Open*
Manuscript ID	bmjopen-2022-066129
Article Type:	Protocol
Date Submitted by the Author:	29-Jun-2022

Complete List of Authors:	Dambi, Jermaine; Faculty of Medicine and Health Sciences, Primary Healthcre Sciences<br>Cowan, F; Centre for Sexual Health and HIV/AIDS Research; Liverpool School of Tropical Medicine<br>Martin, Faith; Coventry University<br>Sibanda, Sharon; Centre for Sexual Health and HIV/AIDS Research<br>Simms, Victoria; London School of Hygiene & Tropical Medicine<br>Willis, Nicola; AFRICAID ZVANDIRI<br>Bernays , Sarah; London School of Hygiene & Tropical Medicine; University of Sydney<br>Mavhu, Webster; Centre for Sexual Health and HIV/AIDS Research; Liverpool School of Tropical Medicine

Keywords:	Child & adolescent psychiatry < PSYCHIATRY, MENTAL HEALTH, SOCIAL MEDICINE

SCHOLARONE™
Manuscripts

Title: A conceptualisation and psychometric evaluation of positive psychological outcome measures used in adolescents and young adults living with HIV: a mixed scoping and systematic review protocol.

Authors: Jermaine M. Dambi [1,§], Frances M. Cowan [2,3], Faith Martin [4], Sharon Sibanda [2], Victoria Simms [5], Nicola Willis [6], Sarah Bernays [5,7], Webster Mavhu [2,3]

Affiliations:

[1] University of Zimbabwe: Rehabilitation Sciences Unit, Faculty of Medicine, and Health Sciences.

PO Box AV 178 Avondale, Harare, Zimbabwe

[2] Centre for Sexual Health and HIV/AIDS Research (CeSHHAR), 4 Bath Harare, Zimbabwe

[3] Liverpool School of Tropical Medicine, Pembroke Pl, Liverpool L3 5QA, United Kingdom

[4] Coventry University, Priory St, Coventry CV1 5FB, United Kingdom

[5] London School of Hygiene & Tropical Medicine, Keppel St, London WC1E 7HT, United Kingdom

[6] AFRICAID ZVANDIRI. 11-12 Stoneridge Way North, Avondale, Harare, Zimbabwe

[7] University of Sydney, Camperdown NSW 2006, Australia

§ Corresponding author

Email addresses:

Jermaine M. Dambi (JMD): jermainedambi@gmail.com

Frances M. Cowan (FMC): frances.cowan@lstmed.ac.uk

Faith Martin (FM): aa6530@coventry.ac.uk

Sharon Sibanda (SS): sharonsibanda07@gmail.com

Victoria Simms (VS): victoria.simms@lshtm.ac.uk

Nicola Willis (NW): nicola@zvandiri.org

Sarah Bernays (SB): sarah.bernays@sydney.edu.au

Webster Mavhu (WM): webster@ceshhar.co.zw

Word count: 2343 words

For peer review only

Abstract

Introduction: The sub-Saharan Africa region bears the greatest burden of HIV in young people. Concomitant mental disorders are common, thus necessitating the integration of mental healthcare into routine HIV care. Consequently, it is necessary to holistically evaluate the mental health of young people with HIV by measuring negative and positive psychological constructs. There has been a proliferation of positive psychological outcome measures, but the evidence of their psychometric robustness is fragmented. This review therefore seeks:

1) identify and map positive psychological outcomes and corresponding measures used in young people living with HIV in sub-Saharan Africa
2) critically appraise the psychometrics of the identified positive psychology outcomes

Methods: This mixed review will be done in two parts; first, a scoping review will map the positive psychological constructs and identify the corresponding outcome measures. The second part will systematically evaluate the psychometric properties of the identified outcomes. Two independent and blinded reviewers will search articles on PubMed, Scopus, Web of Science, Africa- Wide Information, CINAHL, PsychINFO, and Google Scholar. Thereafter, three independent reviewers will screen the retrieved articles. The risk of bias across studies will be evaluated using the COnsensus-based Standards for the selection of health Measurement INstruments (COSMIN) checklist. The quality of the psychometric properties will be rated using the COSMIN checklist and qualitatively synthesised using the modified Grading of Recommendations Assessment, Development, and Evaluation (GRADE) checklist.

Discussion: The proposed systematic review will appraise the collective evidence of the psychometric properties of positive psychological outcomes used in adolescents and young adults living with HIV (AYALHIV). We will glean an initial item bank from robust outcome measures to inform the development of a context-specific outcome measure for AYALHIV in low-resource settings. The outputs will collectively inform the development, implementation, and evaluation of bespoke interventions for AYALHIV.

Registration: PROSEPERO - CRD42022325172.

Keywords: Adolescents; HIV/AIDS; psychometrics; positive psychology; systematic review protocol

sub-Saharan Africa.

For peer review only

Background

HIV remains a global health problem, with low- and middle-income countries disproportionately affected [1]. Further, the burden of HIV in young people in low-resource settings, particularly in the sub-Saharan Africa (SSA) region, remains high [2,3]. Adolescence and early adulthood are challenging developmental stages, with the burden of navigating life challenges often even greater for adolescents and young adults living with HIV (AYALHIV) [1,3,4]. For instance, young persons living with HIV face multiple biopsychosocial challenges, including stigma, negotiating reproductive health, socioeconomic deprivation, violence and other difficulties [1–3,5]. Also, with the increased availability and uptake of highly effective antiretroviral therapy, HIV has evolved into a long-term condition with a concomitant surge in comorbid non-communicable diseases [4,6]. For example, common mental disorders, including anxiety and depression, are prevalent in AYALHIV, with a pooled prevalence of 26.1% (95% CI 18.9–34.8) [3]. However, there is a dearth of integrated programmes combining HIV and mental health care. [1,3,4]. Importantly, many mental health conditions present in adulthood emerge in late adolescence and young adulthood, and effective management at this stage can prevent long-term mental illness [3,4]. Systematic reviews have demonstrated that access to mental healthcare by young people living with HIV is associated with positive outcomes across the treatment continuum, including; increased treatment initiation, increased adherence to care, viral load control, reduced morbidity and mortality and retention in care [1,4].

However, the endpoints of mental healthcare in HIV care have been traditionally conceptualised as improvements in negative psychiatric symptomatology [5,7]. For example, success in psychotherapies is invariably benchmarked against declines in anxiety, depression, post-traumatic stress disorders, and other negative psychological indices [5]. However, focusing on negative indices misses the opportunity to capture the multidimensionality of mental health. A holistic mental health evaluation requires a comprehensive focus on negative and positive mental health constructs [5, 7], and recognition of this has resulted in a shift towards positive psychology, a framework that emphasises increasing human well-being and positive functioning [5]. Positive mental health interventions (PMHIs) are anchored upon the need to improve and evaluate human strengths and capabilities as enshrined in positive outcomes such as self-esteem, resilience, hope, self-worth, social resources, and flourishing [5,7,8]. For instance, studies have shown that people living with a chronic condition (e.g., HIV) develop resilience with time [5,8]. Resilience is defined as the "…*positive psychological, behavioral, and/or social adaptation in the face of stressors and adversities…",* and is a known buffer to stressful life events [8]. The resilience developed in navigating the challenges of living with a chronic condition is potentially transferable into everyday functioning [8]. Positive psychology interventions (e.g., resilience-building approaches) are central to prevention health promotion and act as an entry point to stepped-up care for mental problems in routine HIV care [5].

With the proliferation of PMHIs comes the need to routinely evaluate the clinical endpoints from both the clients' and therapists' perspectives [9]. The patient-reported outcomes revolution is hinged upon assessing treatment success or lack thereof from the patient perspective [7,9]. Patient-reported

For peer review only - http://bmjopen.bmj.com/site/about/guidelines.xhtml

outcomes facilitate patient-clinician communication and clinical decision-making; this is pivotal to patient-centred care [7]. The patient's evaluation of their health, treatment expectations and outcomes are contingent upon the availability of validated and reliable outcome measures [7]. The last few decades have seen a proliferation of positive psychology outcome measures [10]. However, there is limited understanding of the salient positive psychological constructs linked to adolescents and young adults living with HIV (AYALHIV)'s improved well-being and health-related quality of life. Wayant et al. (2021), in their scoping review, mapped 15 positive psychological constructs associated with increased quality of life and survival in adolescents and young people living with cancer [11]. Well-being, personal growth, hope, meaning in life, self-esteem, vitality and optimism were the most cited positive constructs [11]; these constructs are potentially transferable to AYALHIV. Conversely, etiological differences between cancer and HIV could also lead to differences in lived experiences, resulting in differential perceptions in positive psychological constructs [11]. For instance, HIV-related stigma (often associated with issues related to HIV's potential infectiousness) may have a grater impact on mental health functioning in AYALHIV [12] when compared to the effects of cancer-related stigma [13]. It is thus critical to contextualise the impacts of positive psychological outcomes in AYALHIV. Govindasamy et al. (2021) performed a mixed-methods systematic review to explore correlates of well-being among young people living with HIV in SSA to inform econometric evaluations [12]. The review showed that social support, belonging, purpose in life, and self-acceptance optimise well-being in young people living with HIV [12]. Although the review provides essential insights, the sole focus on well-being limits our comprehensive understanding of the spectrum of positive psychological constructs in AYALHIV residing in SSA. There is a need to build on Govindasamy et al.'s work to understand positive psychological constructs in HIV care for AYALHIV holistically. Also, there is a paucity of collective evidence of the psychometric robustness of the positive psychological outcomes used in young people living with HIV. Some of the available generic outcomes may not comprehensively reflect the nuances of living with HIV [12]. The proposed review seeks to answer the following research question(s): what is the collective evidence of the reliability, validity, responsiveness and interpretability [13], of positive psychological outcomes? This mixed review, therefore, seeks to:

1) identify positive psychological outcomes and corresponding outcome measures used in young people living with HIV in SSA
2) critically appraise the psychometric properties of the identified positive psychology outcomes used in AYALHIV
3) map factors associated with the identified positive psychological constructs
4) glean an item bank from outcomes with robust psychometrics

The proposed review will map positive psychological constructs in AYALHIV in SSA, including strengthening the measurement of positive mental health constructs to improve the well-being of this group of young people. The review will also assist in identifying psychometrics that may require further evaluation and systematically identify factors influencing positive psychological function in AYALHIV.

More important, the initial item bank will inform the development of a context-specific positive psychology outcome measure for routine clinical and research use. The resultant outcome measure will

For peer review only - http://bmjopen.bmj.com/site/about/guidelines.xhtml

contribute to the evidence base of the utility of positive psychological interventions in AYALHIV residing in low-income settings.

Methods

Protocol/ registration

This mixed review will be done in two parts; first, a scoping review will map the positive psychological constructs and identify the corresponding outcome measures. The scoping review will be performed per Preferred Reporting Items for Systematic reviews and Meta-Analyses extension for Scoping Reviews (PRISMA-ScR) guidelines - See Additional File 1 [14]. The second part will systematically evaluate the psychometric properties of the identified outcomes; this will be performed and reported according to the Preferred Reporting Items of Systematic Reviews and Meta-Analyses Protocol (PRISMA-P) guidelines - See Additional File 2 [15]. The protocol was registered on the PROSPERO database - CRD42022325172.

Eligibility criteria

The following criterion will be applied in selecting articles:

Study designs/interventions

We will include all quantitative designs, mixed methods and qualitative studies exploring positive pyschological phenomenon in AYALHIV in SSA. Systematic reviews, editorials, case studies and study protocols will be excluded.

Participants/settings

We will analyse all studies reporting on the use and evaluation of positive psychological constructs in AYALHIV in SSA across all settings. For this review, the age range for AYALHIV is 13-24 years. We focus on AYALHIV as it is the group with the greatest burden of HIV globally [12]. We anticipate that some studies will contain data on AYALHIV and other age bands (e.g., children, middle-aged adults). In such cases, an article will be considered for review if the average age is within the 13-24 years range or if over 50% of the participants are AYALHIV.

Language

We will restrict the analysis to articles published in English. We do not have resources to analyse articles published in other languages.

Information sources

Peer-reviewed articles will be searched/retrieved from these electronic databases; PubMed, Scopus, Web of Science, Africa- Wide Information, CINAHL, PsychINFO, and Google Scholar. Databases will be searched from inception through June 2022. Where only an abstract is available online, an attempt to contact the lead author will be made requesting the full article to ensure literature saturation. The article will be excluded from the review if there is no response in two weeks following three email reminders. We will also review grey literature by using the Google Scholar search engine to search potential databases such as university databases and conference proceedings, among others, for articles. For completeness, we will also perform both backwards and forward searches of the reference

For peer review only

lists of identified articles and databases, respectively. Finally, we will also contact experts implementing PMHIs to check for articles we may have missed using the proposed search strategy.

Search strategy

As an illustration, articles in CINAHL will be searched using the AND Boolean logic operators, i.e. 1 AND 6 AND 9 AND 12 (Table 1). **<Insert Table 1 here>**

Table 1: CINAL search strategy

Search #	Query
**1**	**adolescen* OR juvenile* OR teen* OR youth* OR "young person*" OR "young people" OR Adolescence OR Young Adult**
2	Hiv OR hiv-1* OR hiv-2*OR hiv1 OR hiv2 OR "HIV infect*" OR "human immunodeficiency virus" OR "human immunodeficiency virus" OR "human immuno-deficiency virus" OR "human immune-deficiency virus" OR "acquired immunodeficiency syndrome" OR "acquired immunodeficiency syndrome" OR "acquired immuno-deficiency syndrome" OR "acquired immune-deficiency syndrome" OR HIV Infections OR Human Immunodeficiency Virus
3	"human immun*" AND "deficiency virus"
4	hiv or aids or acquired human immunodeficiency syndrome or human immunodeficiency virus OR HIV/AIDS
5	"acquired immun*" AND "deficiency syndrome"
**6**	**2 OR 3 OR 4 0R 5**
7	hope* OR optimis* OR resilien* OR cope OR coping OR gratitude OR grateful OR happiness OR joy OR gladness OR satisf* OR "self efficacy" OR self-efficacy OR content*OR wellbeing OR well-being OR "self acceptance" OR self-acceptance OR "self esteem" OR self-esteem OR "self concept" OR self-concept OR "self confidence" OR self-confidence OR "self perception" OR self-perception OR "self worth" OR self-worth OR "personal growth" OR tranquil* OR perseverance OR vitality OR meaning OR "social* inclus*" OR "social participation" OR "social engagement" OR "social support" OR "self care" OR self-care OR "positive attitude" OR "positive thinking" OR "positive mindset" OR mindfulness OR empower*OR love OR spiritual* OR "community integration" OR "community participation" OR humour OR dignity OR pleasure OR creativ* OR transcend* OR goal* OR Psychological Well-Being OR Hope OR Optimism OR Coping OR Human Dignity OR Hardiness OR Adaptation, Psychological OR Happiness OR Personal Satisfaction OR Self Efficacy OR Self Concept OR Social Inclusion OR Social Participation OR Self Care OR Mindfulness OR Empowerment OR Love OR Spirituality OR Social Networks OR Pleasure OR Creativeness OR Self Transcendence OR (Goals and Objectives)
8	hope* OR optimis* OR optimism OR resilience OR resilien* OR resilient OR cope OR coping OR gratitude OR grateful OR happiness OR joy OR gladness OR (life satisfaction) OR satisfaction OR satisf* OR "self efficacy" OR self-efficacy OR content OR contentment OR content*OR wellbeing OR well-being OR "self acceptance" OR self-acceptance OR "self esteem" OR self-esteem OR "self concept" OR self-concept OR "self confidence" OR self-confidence OR "self perception" OR self-perception OR "self worth" OR self-worth OR "personal growth" OR tranquillity OR tranquil* OR perseverance OR vitality OR meaning OR "social* inclus*" OR "social participation" OR "social engagement" OR "social support" OR "self care" OR self-care OR "positive attitude" OR "positive thinking" OR "positive mindset" OR mindfulness OR empowerment OR empower*OR love OR spirituality OR spiritual* OR "community integration" OR "community participation" OR humour OR dignity OR pleasure OR creativ* OR transcend* OR goal* OR Psychological Well-Being OR Hope OR Optimism OR Coping OR Human Dignity OR Hardiness OR Adaptation, Psychological OR Happiness OR Personal Satisfaction OR Self Efficacy OR Self Concept OR Social Inclusion OR Social Participation OR Self Care OR Mindfulness OR Empowerment OR Love OR Spirituality OR Social Networks OR Pleasure OR Creativeness OR Self Transcendence OR (Goals and Objectives)
**9**	**7 OR 8**
10	Angola or Benin or Botswana or Burkina Faso or Burundi or Cameroon or Cape Verde or Central African Republic or CHAD or Comoros or Congo or Congo Democratic Republic or Djibouti or Equatorial Guinea or Eritrea or Ethiopia or Gabon or Gambia or Ghana or Guinea or Guinea-Bissau or Cote d'Ivoire or Ivory Coast or Kenya or Lesotho or Liberia or Madagascar or Malawi or Mali or Mozambique or Namibia or Niger or Nigeria or Sao tome and Principe or Rwanda or Senegal or Seychelles or Sierra Leone or Somalia or South Africa or South Sudan or Sudan or Swaziland or Tanzania or Togo or Uganda or Zambia or Zimbabwe
11	'Africa, South of the Sahara' OR "sub-Saharan Africa"

For peer review only - http://bmjopen.bmj.com/site/about/guidelines.xhtml

12	10 OR 11

Adapted from [18]

Data management

Retrieved articles will be imported into the Mendeley reference manager, which is password protected. The articles will also be synchronised onto Mendeley and Dropbox cloud storage platforms and backed up onto a password-encrypted external hard drive. All collaborators will have full access/administrative privileges to the shared Dropbox folder for the present systematic review. A trail/history of the electronic searches will also be saved on users' PubMed, Scopus, and EBSCOhost accounts. We will also print the summaries of all the searches to enhance the data capturing of the search records.

Data collection process

Two researchers will independently search articles using a pre-defined search strategy. The lead author (JMD) will then import the searches into Mendeley and remove duplicates. Afterwards, another set of independent researchers (SS & WM) will screen the articles by title and abstract using Rayyan software [16]. JMD and SS will then perform backwards and forward citation searches to identify other potential articles. Two senior researchers (FMC & WM) will review the list of identified articles afterwards to check for the completeness of the searches. Once searches are finalised, two researchers (FM and NW) will retrieve the full articles and extract the following information per study; research setting and design, study sample, participants' demographics, mode of administration, and evaluate the risk of bias per study in Microsoft Excel. VS and SB will then evaluate the quality of content validity. Afterwards, JMD and FM will synthesise the level of evidence of the content validity across retrieved outcomes, which will be cross-checked by FMC and WM. Disagreements during data extraction will be resolved through consensus, and more senior researchers (FMC & WM) will make the final decisions if any impasses occur.

Outcomes and prioritisation

For this review, conceptualisation of positive psychological constructs and the psychometric properties of the identified outcomes will be the primary outcome measures with clinical utility being the secondary outcome. See Table 2 for operational definitions of psychometric properties for the present review [17,18].

< Insert Table 2 here>

Table 2: Operational definitions of psychometric properties [17,18]

Term			Definition
**Domain**	**Measurement property**	**Aspect of a measurement property**	
**Reliability**			The degree to which the measurement is free from measurement error
**Reliability (extended definition)**			The extent to which scores for patients who have not changed are the same for repeated measurement under several conditions: e.g., using different sets of items from the same PROM (internal consistency); over time (test-retest); by different persons on the same occasion (inter-rater); or by the same persons (i.e., raters or responders) on different occasions (intra-rater)

For peer review only

	**Internal consistency**		The degree of the interrelatedness among the items
	**Reliability**		The proportion of the total variance in the measurements which is due to 'true'† differences between patients
	**Measurement error**		The systematic and random error of a patient's score that is not attributed to true changes in the construct to be measured
**Validity**			The degree to which a PROM measures the construct(s) it purports to measure
	**Content validity**		The degree to which the content of a PROM is an adequate reflection of the construct to be measured
		**Face validity**	The degree to which (the items of) a PROM indeed looks as though they are an adequate reflection of the construct to be measured
	**Construct validity**		The degree to which the scores of a PROM are consistent with hypotheses *(for instance with regard to internal relationships, relationships to scores of other instruments, or differences between relevant groups)* based on the assumption that the PROM validly measures the construct to be measured
		**Structural validity**	The degree to which the scores of a PROM are an adequate reflection of the dimensionality of the construct to be measured
		**Hypotheses testing**	Item construct validity
		**Cross-cultural validity**	The degree to which the performance of the items on a translated or culturally adapted PROM are an adequate reflection of the performance of the items of the original version of the PROM
	**Criterion validity**		The degree to which the scores of a PROM are an adequate reflection of a 'gold standard'
**Responsiveness**			The ability of a PROM to detect change over time in the construct to be measured
	**Responsiveness**		Idem responsiveness
**Interpretability***			Interpretability is the degree to which one can assign qualitative meaning - that is, clinical or commonly understood connotations – to a PROM's quantitative scores or change in scores.

† The word 'true' must be seen in the context of the CTT, which states that any observation is composed of two components – a true score and error associated with the observation. 'True' is the average score that would be obtained if the scale were given an infinite number of times. It refers only to the consistency of the score, and not to its accuracy.
** Interpretability is not considered a measurement property, but an important characteristic of a measurement instrument*

Risk of bias individual studies

We will use the revised COnsensus-based Standards for the selection of health Measurement INstruments (COSMIN) checklist to assess the risk of bias across the retrieved studies [17,18]. The COSMIN methodology consists of three steps. The checklist consists of methodological benchmarks for ten (10) psychometric properties, which are categorised into three major groups, i.e. content validity (e.g. patient-reported outcome measure development), the internal structure (e.g. structural validity),

For peer review only

and other psychometrical properties (e.g. criterion validity) [17,18]. Each psychometric property is rated using a pre-set criterion and using the principle of "worse score counts", the lowest rating is ascribed as the overall methodological quality rating [18]. Methodological quality is rated on a four-point Likert scale, i.e. "inadequate", "doubtful", "adequate", and "very good"; the higher the rating, the lower the risk of bias [17,18]. We anticipate that not all details may be recorded for the retrieved articles, especially for studies whose primary aim was not psychometric evaluation. We will, therefore, contact the correspondent authors to achieve the most truthful rating of the psychometric property to decrease bias at analysis.

Psychometric properties and data extraction

The quality of psychometrical properties will be evaluated using an updated, hybrid checklist based on previous work by Terwee et al. [19] and Prinsen et al. [20] (See Table 3). Each psychometric property will be rated as; sufficient (+), insufficient (–), or indeterminate (?) [17]. Positive ratings represent high-quality psychometrics [17]. ***< Insert Table 3 here>***

Table 3: Updated criteria for good measurement properties [15,16]

Measurement property	Rating[1]	Criteria
Structural validity	+	**CTT:**<br>CFA: CFI or TLI or comparable measure >0.95 OR RMSEA <0.06 OR SRMR <0.08[2]<br>**IRT/Rasch**:<br>No violation of unidimensionality[3]: CFI or TLI or comparable measure >0.95 OR RMSEA <0.06 OR SRMR <0.08<br>*AND*<br>no violation of local independence: residual correlations among the items after controlling for the dominant factor < 0.20 OR Q3's < 0.37<br>*AND*<br>no violation of monotonicity: adequate looking graphs OR item scalability >0.30<br>*AND*<br>adequate model fit:<br>IRT: χ^2 >0.01<br>Rasch: infit and outfit mean squares ≥ 0.5 and ≤ 1.5 OR Z-standardised values > -2 and <2
	?	CTT: Not all information for '+' reported<br>IRT/Rasch: Model fit not reported
	–	Criteria for '+' not met
Internal consistency	+	At least low evidence[4] for sufficient structural validity[5] AND Cronbach's alpha(s) ≥ 0.70 for each unidimensional scale or subscale[6]
	?	Criteria for "At least low evidence[4] for sufficient structural validity[5]" not met
	–	At least low evidence[4] for sufficient structural validity[5] AND Cronbach's alpha(s) < 0.70 for each unidimensional scale or subscale[6]
	+	ICC or weighted Kappa ≥ 0.70

For peer review only

Reliability	?	ICC or weighted Kappa not reported
	–	ICC or weighted Kappa < 0.70
Measurement error	+	SDC or LoA < MIC[5]
	?	MIC not defined SDC or LoA
	–	> MIC[5]
Hypotheses testing for construct validity	+	The result is in accordance with the hypothesis[7]
	?	No hypothesis defined (by the review team)
	–	The result is not in accordance with the hypothesis[7]
Cross-cultural validity\measurement invariance	+	No important differences found between group factors (such as age, gender, language) in multiple group factor analysis OR no important DIF for group factors (McFadden's R^2 < 0.02)
	?	No multiple group factor analysis OR DIF analysis performed
	–	Important differences between group factors OR DIF was found
Criterion validity	+	Correlation with gold standard ≥ 0.70 OR AUC ≥ 0.70
	?	Not all information for '+' reported
	–	Correlation with gold standard < 0.70 OR AUC < 0.70
Responsiveness	+	The result is in accordance with the hypothesis[7] OR AUC ≥ 0.70
	?	No hypothesis defined (by the review team)
	–	The result is not in accordance with the hypothesis[7] OR AUC < 0.70

Key: AUC = area under the curve, CFA = confirmatory factor analysis, CFI = comparative fit index, CTT = classical test theory, DIF = differential item functioning, ICC = intraclass correlation coefficient, IRT = item response theory, LoA = limits of agreement, MIC = minimal important change, RMSEA: Root Mean Square Error of Approximation, SEM = Standard Error of Measurement, SDC= smallest detectable change, SRMR: Standardized Root Mean Residuals, TLI = Tucker-Lewis index.

Best evidence synthesis

Firstly, we will develop a conceptual framework for synthesising both qualitative and quantitative studies retrieved. We will apply a narrative synthesis to map the key “themes/constructs” emerging from the scoping review. The conceptual framework/model will map the constructs and subsequently guide the psychometric evaluation. The collective evidence per psychometric property per outcome will be synthesised using the modified Grading of Recommendations Assessment, Development, and Evaluation (GRADE) checklist [21], as outlined in Table 4. The modified GRADE will be used to collate the risk of bias assessment results and the quality of psychometric ratings to qualitatively synthesise/summarise the quality of evidence per psychometric property across studies. The quality of evidence per psychometrical property will be classified as very low, low, moderate or high [21].

<Insert Table 4 here>

For peer review only

Table 4: GRADE checklist- best evidence synthesis [22]

Quality level	Definition/criterion
High	We are very confident that the true measurement property lies close to that of the estimate of the measurement property
Moderate	We are moderately confident in the measurement property estimate: the true measurement property is likely to be close to the estimate of the measurement property, but there is a possibility that it is substantially different
Low	Our confidence in the measurement property estimate is limited: the true measurement property may be substantially different from the estimate of the measurement property
Very low	We have very little confidence in the measurement property estimate: the true measurement property is likely to be substantially different from the estimate of the measurement property

Patient and Public Involvement statement

We will work collaboratively with AYALHIV during data collection and dissemination. AYALHIV representatives previously trained and involved in systematic reviews will assist with article screening. We will co-create the dissemination plans; for instance, young persons with lived experiences will be involved in co-developing output animation and contribute to the project blogs, amongst other dissemination activities.

Discussion

The proposed systematic review will map and appraise the collective evidence of the psychometric robustness of positive psychological outcomes used in AYALHIV. The proposed review builds on recommendations of systematic reviews on the need to measure positive psychological constructs across diverse populations objectively. This is important given the need to use valid and reliable outcomes in understanding the positive effects of living with HIV. The review will also assist in identifying psychometrically-robust outcomes to inform an item bank to adapt a context-specific outcome measure for AYALHIV in low-resource settings. Also, the review will systematically identify factors influencing well-being in AYALHIV. The outputs will collectively inform the development, implementation, and evaluation of bespoke positive mental health interventions for AYALHIV.

Abbreviations

AYALHIV- adolescents and young adults living with HIV; CINAHL- Cumulative Index of Nursing and Allied Health Literature; COSMIN - COnsensus-based Standards for the selection of health Measurement Instruments; GRADE- Grading of Recommendations Assessment, Development, and Evaluation; HRQoL - health-related quality of life; PRISMA-P - Preferred Reporting Items of Systematic Reviews and Meta-Analyses Protocol; NCD- non-communicable disease.

Declarations

Ethics approval and consent to participate

Not applicable.

Consent for publication

Not applicable.

For peer review only

Availability of data and materials
Not applicable for a review protocol. However, all relevant data/materials, including data collection tools will be submitted as supplementary files during the publication of the full review.

Competing interests
All the authors declare no competing interests.

Funding
This research was funded in whole or in part by Templeton World Charity Foundation, Inc. [TWC-2021-20629]. For the purpose of open access, the author has applied a CC-BY public copyright license to any author accepted manuscript version arising from this submission. The funder had no role in study design, data collection, data analysis, data interpretation, or writing of the report.

Authors' contributions
JMD was primarily responsible for protocol writing. JMD, FMC, FM, SS, VC, NW, SB and WM were involved in the conceptualisation of the study and editing all protocol manuscript versions. JMD will search the literature and data management. FMC, FM, SS, VC, NW, SB and WM will be responsible for article screening and quality assurance, data extraction and qualitative synthesis.

Acknowledgements
We would like to acknowledge Alison Derbyshire for the assistance with refining the search strategies.

Author's ORCID information
- Jermaine M. Dambi: https://orcid.org/0000-0002-2446-7903
- Frances M. Cowan: https://orcid.org/0000-0003-3087-4422
- Faith Martin: https://orcid.org/0000-0002-0141-1210
- Sharon Sibanda: https://orcid.org/0000-0002-6595-4097
- Victoria Simms: https://orcid.org/0000-0002-4897-458X
- Nicola Willis: https://orcid.org/0000-0003-0452-0196
- Sarah Bernays: https://orcid.org/0000-0001-7628-8408
- Webster Mavhu: https://orcid.org/0000-0003-1881-4398

References

1 Bhana A, Abas MA, Kelly J, et al. Mental health interventions for adolescents living with HIV or affected by HIV in low- and middle-income countries: systematic review. BJPsych Open 2020;6:1–15. doi:10.1192/bjo.2020.67

2 Dow DE, Mmbaga BT, Turner EL, et al. Building resilience: a mental health intervention for Tanzanian youth living with HIV. AIDS Care 2018;30:12–20. doi:10.1080/09540121.2018.1527008

3 Ayano G, Demelash S, Abraha M, et al. The prevalence of depression among adolescent with HIV/AIDS: a systematic review and meta-analysis. AIDS Res Ther 2021;18:1–10. doi:10.1186/s12981-021-00351-1

4 Bhana A, Kreniske P, Pather A, et al. Interventions to address the mental health of adolescents and young adults living with or affected by HIV: state of the evidence. J Int AIDS Soc 2021;24:87–99. doi:10.1002/jia2.25713

5 Bolier L, Haverman M, Westerhof GJ, et al. Positive psychology interventions: A meta-analysis of randomised controlled studies. BMC Public Health 2013;13. doi:10.1186/1471-2458-13-119

6 Adeyemi O, Lyons M, Njim T, et al. Integration of non-communicable disease and HIV/AIDS management: A review of healthcare policies and plans in East Africa. BMJ Glob Heal 2021;6:1–9. doi:10.1136/bmjgh-2020-004669

7 Bristowe K, Murtagh FEM, Clift P, et al. The development and cognitive testing of the positive outcomes HIV PROM: A brief novel patient-reported outcome measure for adults living with HIV. Health Qual Life Outcomes 2020;18:1–10. doi:10.1186/s12955-020-01462-5

8 Dulin AJ, Dale SK, Earnshaw VA, et al. Resilience and HIV: a review of the definition and study of resilience. AIDS Care - Psychol Socio-Medical Asp AIDS/HIV 2018;30:S6–17. doi:10.1080/09540121.2018.1515470

For peer review only

9 Kall M, Marcellin F, Harding R, et al. Patient-reported outcomes to enhance person-centred HIV care. Lancet HIV 2020;7:e59–68. doi:10.1016/S2352-3018(19)30345-5

10 Stoner CR, Stansfeld J, Orrell M, et al. The development of positive psychology outcome measures and their uses in dementia research: A systematic review. Dementia 2017;18:2085–106. doi:10.1177/1471301217740288

11 Wayant C, Manquen J, Wendelbo H, et al. Identification of Evidence for Key Positive Psychological Constructs in Pediatric and Adolescent/Young Adult Patients with Cancer: A Scoping Review. J Adolesc Young Adult Oncol 2021;10:247–59. doi:10.1089/jayao.2020.0184

12 Govindasamy D, Seeley J, Olaru ID, et al. Informing the measurement of wellbeing among young people living with HIV in sub-Saharan Africa for policy evaluations: A mixed-methods systematic review. Health Qual Life Outcomes 2020;18. doi:10.1186/s12955-020-01352-w

13 Munn Z, Stern C, Aromataris E, et al. What kind of systematic review should i conduct? A proposed typology and guidance for systematic reviewers in the medical and health sciences. BMC Med Res Methodol 2018;18:1–9. doi:10.1186/s12874-017-0468-4

14 Tricco AC, Lillie E, Zarin W, et al. PRISMA Extension for Scoping Reviews (PRISMA-ScR): Checklist and Explanation. Ann Intern Med 2018;169:467–73. doi:10.7326/M18-0850

15 Page MJ, Mckenzie JE, Bossuyt PM, et al. The PRISMA 2020 statement : an updated guideline for reporting systematic reviews Systematic reviews and Meta-Analyses. BMJ 2021;372. doi:10.1136/bmj.n71

16 Ouzzani M, Hammady H, Fedorowicz Z, et al. Rayyan-a web and mobile app for systematic reviews. Syst Rev 2016;5:1–10. doi:10.1186/s13643-016-0384-4

17 Vet LBMHCW De, Patrick CACPDL, Bouter JALM, et al. COSMIN Risk of Bias checklist for systematic reviews of Patient- Reported Outcome Measures. Qual Life Res 2018;27:1171–9. doi:10.1007/s11136-017-1765-4

18 Terwee CB, Prinsen CAC, Chiarotto A, et al. COSMIN methodology for evaluating the content validity of patient-reported outcome measures: a Delphi study. Qual Life Res 2018;27:1159–70. doi:10.1007/s11136-018-1829-0

19 Terwee CB, Bot SDM, de Boer MR, et al. Quality criteria were proposed for measurement properties of health status questionnaires. J Clin Epidemiol 2007;60:34–42. doi:10.1016/j.jclinepi.2006.03.012

20 Prinsen CAC, Vohra S, Rose MR, et al. How to select outcome measurement instruments for outcomes included in a “ Core Outcome Set ” – a practical guideline. Trials 2016;17:1–10. doi:10.1186/s13063-016-1555-2

21 Zhang Y, Alonso-Coello P, Guyatt GH, et al. GRADE Guidelines: 19. Assessing the certainty of evidence in the importance of outcomes or values and preferences—Risk of bias and indirectness. J Clin Epidemiol 2019;111:94–104. doi:10.1016/j.jclinepi.2018.01.013

For peer review only

Preferred Reporting Items for Systematic reviews and Meta-Analyses extension for Scoping Reviews (PRISMA-ScR) Checklist

SECTION	ITEM	PRISMA-ScR CHECKLIST ITEM	REPORTED ON PAGE #
**TITLE**			
Title	1	Identify the report as a scoping review.	1
**ABSTRACT**			
Structured summary	2	Provide a structured summary that includes (as applicable): background, objectives, eligibility criteria, sources of evidence, charting methods, results, and conclusions that relate to the review questions and objectives.	2
**INTRODUCTION**			
Rationale	3	Describe the rationale for the review in the context of what is already known. Explain why the review questions/objectives lend themselves to a scoping review approach.	3-5
Objectives	4	Provide an explicit statement of the questions and objectives being addressed with reference to their key elements (e.g., population or participants, concepts, and context) or other relevant key elements used to conceptualize the review questions and/or objectives.	4
**METHODS**			
Protocol and registration	5	Indicate whether a review protocol exists; state if and where it can be accessed (e.g., a Web address); and if available, provide registration information, including the registration number.	5
Eligibility criteria	6	Specify characteristics of the sources of evidence used as eligibility criteria (e.g., years considered, language, and publication status), and provide a rationale.	5
Information sources*	7	Describe all information sources in the search (e.g., databases with dates of coverage and contact with authors to identify additional sources), as well as the date the most recent search was executed.	5
Search	8	Present the full electronic search strategy for at least 1 database, including any limits used, such that it could be repeated.	6-7
Selection of sources of evidence†	9	State the process for selecting sources of evidence (i.e., screening and eligibility) included in the scoping review.	6
Data charting process‡	10	Describe the methods of charting data from the included sources of evidence (e.g., calibrated forms or forms that have been tested by the team before their use, and whether data charting was done independently or in duplicate) and any processes for obtaining and confirming data from investigators.	7
Data items	11	List and define all variables for which data were sought and any assumptions and simplifications made.	7
Critical appraisal of individual sources of evidence§	12	If done, provide a rationale for conducting a critical appraisal of included sources of evidence; describe the methods used and how this information was used in any data synthesis (if appropriate).	10

For peer review only - http://bmjopen.bmj.com/site/about/guidelines.xhtml

SECTION	ITEM	PRISMA-ScR CHECKLIST ITEM	REPORTED ON PAGE #
Synthesis of results	13	Describe the methods of handling and summarizing the data that were charted.	8
**RESULTS**			
Selection of sources of evidence	14	Give numbers of sources of evidence screened, assessed for eligibility, and included in the review, with reasons for exclusions at each stage, ideally using a flow diagram.	n/a
Characteristics of sources of evidence	15	For each source of evidence, present characteristics for which data were charted and provide the citations.	N/A
Critical appraisal within sources of evidence	16	If done, present data on critical appraisal of included sources of evidence (see item 12).	N/A
Results of individual sources of evidence	17	For each included source of evidence, present the relevant data that were charted that relate to the review questions and objectives.	N/A
Synthesis of results	18	Summarize and/or present the charting results as they relate to the review questions and objectives.	N/A
**DISCUSSION**			
Summary of evidence	19	Summarize the main results (including an overview of concepts, themes, and types of evidence available), link to the review questions and objectives, and consider the relevance to key groups.	9
Limitations	20	Discuss the limitations of the scoping review process.	N/A
Conclusions	21	Provide a general interpretation of the results with respect to the review questions and objectives, as well as potential implications and/or next steps.	N/A
**FUNDING**			
Funding	22	Describe sources of funding for the included sources of evidence, as well as sources of funding for the scoping review. Describe the role of the funders of the scoping review.	9

JBI = Joanna Briggs Institute; PRISMA-ScR = Preferred Reporting Items for Systematic reviews and Meta-Analyses extension for Scoping Reviews.

* Where *sources of evidence* (see second footnote) are compiled from, such as bibliographic databases, social media platforms, and Web sites.

† A more inclusive/heterogeneous term used to account for the different types of evidence or data sources (e.g., quantitative and/or qualitative research, expert opinion, and policy documents) that may be eligible in a scoping review as opposed to only studies. This is not to be confused with *information sources* (see first footnote).

‡ The frameworks by Arksey and O'Malley (6) and Levac and colleagues (7) and the JBI guidance (4, 5) refer to the process of data extraction in a scoping review as data charting.

§ The process of systematically examining research evidence to assess its validity, results, and relevance before using it to inform a decision. This term is used for items 12 and 19 instead of "risk of bias" (which is more applicable to systematic reviews of interventions) to include and acknowledge the various sources of evidence that may be used in a scoping review (e.g., quantitative and/or qualitative research, expert opinion, and policy document).

From: Tricco AC, Lillie E, Zarin W, O'Brien KK, Colquhoun H, Levac D, et al. PRISMA Extension for Scoping Reviews (PRISMAScR): Checklist and Explanation. Ann Intern Med. 2018;169:467–473. doi: 10.7326/M18-0850.

For peer review only - http://bmjopen.bmj.com/site/about/guidelines.xhtml

PRISMA-P 2015 Checklist

This checklist has been adapted for use with protocol submissions to *Systematic Reviews* from Table 3 in Moher D et al: Preferred reporting items for systematic review and meta-analysis protocols (PRISMA-P) 2015 statement. *Systematic Reviews* 2015 **4**:1

Section/topic	#	Checklist item	Information reported: Yes	Information reported: No	Line number(s)
**ADMINISTRATIVE INFORMATION**					
**Title**					
Identification	1a	Identify the report as a protocol of a systematic review	☒	☐	1
Update	1b	If the protocol is for an update of a previous systematic review, identify as such	☐	☒	N/A
**Registration**	2	If registered, provide the name of the registry (e.g., PROSPERO) and registration number in the Abstract	☒	☐	5
**Authors**					
Contact	3a	Provide name, institutional affiliation, and e-mail address of all protocol authors; provide physical mailing address of corresponding author	☒	☐	1
Contributions	3b	Describe contributions of protocol authors and identify the guarantor of the review	☒	☐	12
**Amendments**	4	If the protocol represents an amendment of a previously completed or published protocol, identify as such and list changes; otherwise, state plan for documenting important protocol amendments	☐	☒	N/A
**Support**					
Sources	5a	Indicate sources of financial or other support for the review	☒	☐	12
Sponsor	5b	Provide name for the review funder and/or sponsor	☒	☐	12
Role of sponsor/funder	5c	Describe roles of funder(s), sponsor(s), and/or institution(s), if any, in developing the protocol	☒	☐	12
**INTRODUCTION**					
**Rationale**	6	Describe the rationale for the review in the context of what is already known	☒	☐	3-5
**Objectives**	7	Provide an explicit statement of the question(s) the review will address with reference to participants, interventions, comparators, and outcomes (PICO)	☒	☐	4
**METHODS**					

For peer review only

2

Section/topic	#	Checklist item	Information reported Yes	Information reported No	Line number(s)
**Eligibility criteria**	8	Specify the study characteristics (e.g., PICO, study design, setting, time frame) and report characteristics (e.g., years considered, language, publication status) to be used as criteria for eligibility for the review	☒	☐	5
**Information sources**	9	Describe all intended information sources (e.g., electronic databases, contact with study authors, trial registers, or other grey literature sources) with planned dates of coverage	☒	☐	5-6
**Search strategy**	10	Present draft of search strategy to be used for at least one electronic database, including planned limits, such that it could be repeated	☒	☐	6-7
STUDY RECORDS					
Data management	11a	Describe the mechanism(s) that will be used to manage records and data throughout the review	☒	☐	7
Selection process	11b	State the process that will be used for selecting studies (e.g., two independent reviewers) through each phase of the review (i.e., screening, eligibility, and inclusion in meta-analysis)	☒	☐	7
Data collection process	11c	Describe planned method of extracting data from reports (e.g., piloting forms, done independently, in duplicate), any processes for obtaining and confirming data from investigators	☒	☐	7
**Data items**	12	List and define all variables for which data will be sought (e.g., PICO items, funding sources), any pre-planned data assumptions and simplifications	☒	☐	7-9
**Outcomes and prioritization**	13	List and define all outcomes for which data will be sought, including prioritization of main and additional outcomes, with rationale	☒	☐	7
**Risk of bias in individual studies**	14	Describe anticipated methods for assessing risk of bias of individual studies, including whether this will be done at the outcome or study level, or both; state how this information will be used in data synthesis	☒	☐	8-9
DATA					
**Synthesis**	15a	Describe criteria under which study data will be quantitatively synthesized	☐	☒	N/A
	15b	If data are appropriate for quantitative synthesis, describe planned summary measures, methods of handling data, and methods of combining data from studies, including any planned exploration of consistency (e.g., I^2, Kendall's tau)	☐	☒	N/A
	15c	Describe any proposed additional analyses (e.g., sensitivity or subgroup analyses, meta-regression)	☐	☒	N/A
	15d	If quantitative synthesis is not appropriate, describe the type of summary planned	☒	☐	10
**Meta-bias(es)**	16	Specify any planned assessment of meta-bias(es) (e.g., publication bias across studies, selective reporting within studies)	☐	☒	N/A
**Confidence in cumulative evidence**	17	Describe how the strength of the body of evidence will be assessed (e.g., GRADE)	☒	☐	10

For peer review only

BioMed Central The Open Access Publisher

BMJ Open

A conceptualisation and psychometric evaluation of positive psychological outcome measures used in adolescents and young adults living with HIV: a mixed scoping and systematic review protocol.

Field	Value
Journal:	*BMJ Open*
Manuscript ID	bmjopen-2022-066129.R1
Article Type:	Protocol
Date Submitted by the Author:	01-Sep-2022

Field	Value
Complete List of Authors:	Dambi, Jermaine; University of Zimbabwe Faculty of Medicine, Primary Healthcare Sciences<br>Cowan, F; Centre for Sexual Health and HIV/AIDS Research; Liverpool School of Tropical Medicine<br>Martin, Faith; Coventry University<br>Sibanda, Sharon; Centre for Sexual Health and HIV/AIDS Research<br>Simms, Victoria; London School of Hygiene & Tropical Medicine<br>Willis, Nicola; AFRICAID ZVANDIRI<br>Bernays , Sarah; London School of Hygiene & Tropical Medicine; University of Sydney<br>Mavhu, Webster; Centre for Sexual Health and HIV/AIDS Research; Liverpool School of Tropical Medicine

Field	Value
**Primary Subject Heading**:	Mental health
Secondary Subject Heading:	HIV/AIDS, Mental health
Keywords:	Child & adolescent psychiatry < PSYCHIATRY, MENTAL HEALTH, SOCIAL MEDICINE

SCHOLARONE™ Manuscripts

Title: A conceptualisation and psychometric evaluation of positive psychological outcome measures used in adolescents and young adults living with HIV: a mixed scoping and systematic review protocol

Authors: Jermaine M. Dambi [1, §], Frances M. Cowan [2,3], Faith Martin [4], Sharon Sibanda [2], Victoria Simms [5], Nicola Willis [6], Sarah Bernays [5,7], Webster Mavhu [2,3]

Affiliations:

[1] University of Zimbabwe: Rehabilitation Sciences Unit, Faculty of Medicine and Health Sciences, PO Box AV 178 Avondale, Harare, Zimbabwe

[2] Centre for Sexual Health and HIV/AIDS Research (CeSHHAR), 4 Bath Road, Harare, Zimbabwe

[3] Liverpool School of Tropical Medicine, Pembroke Pl, Liverpool L3 5QA, United Kingdom

[4] Coventry University, Priory St, Coventry CV1 5FB, United Kingdom

[5] London School of Hygiene & Tropical Medicine, Keppel St, London WC1E 7HT, United Kingdom

[6] AFRICAID ZVANDIRI, 11-12 Stoneridge Way North, Avondale, Harare, Zimbabwe

[7] University of Sydney, Camperdown NSW 2006, Australia

§ Corresponding author

Email addresses:

Jermaine M. Dambi (JMD): jermainedambi@gmail.com

Frances M. Cowan (FMC): frances.cowan@lstmed.ac.uk

Faith Martin (FM): aa6530@coventry.ac.uk

Sharon Sibanda (SS): sharonsibanda07@gmail.com

Victoria Simms (VS): victoria.simms@lshtm.ac.uk

Nicola Willis (NW): nicola@zvandiri.org

Sarah Bernays (SB): sarah.bernays@sydney.edu.au

Webster Mavhu (WM): webster@ceshhar.co.zw

Word count: 2 780 words

For peer review only

Abstract

Introduction: Sub-Saharan Africa bears the greatest burden of HIV. Concomitant mental disorders are common, necessitating the integration of mental healthcare into routine HIV care. Consequently, it is necessary to holistically evaluate the mental health of adolescents and young adults living with HIV (AYALHIV, 10-24 years old) by measuring negative and positive psychological constructs (e.g., anxiety and self-acceptance, respectively). There has been a proliferation of positive psychological outcome measures, but the evidence of their psychometric robustness is fragmented. This review, therefore, seeks to:

1) identify positive psychological outcomes used in AYALHIV in sub-Saharan Africa and map the constructs onto corresponding measures
2) critically appraise the psychometrics of the identified outcomes

Methods and analysis: This mixed review will be done in two parts. Firstly, a scoping review will identify positive psychological outcomes and map them onto corresponding outcome measures. Subsequently, we will systematically evaluate the psychometric properties of the outcomes identified from the scoping review. Independent and blinded reviewers will search articles in PubMed, Scopus, Web of Science, Africa-Wide Information, CINAHL, PsychINFO, and Google Scholar from inception through September 30, 2022. Thereafter, separate independent reviewers will screen the retrieved articles. We will apply a narrative synthesis to map the key constructs emerging from the scoping review. For the systematic review, the risk of bias across studies will be evaluated using the COnsensus-based Standards for the selection of health Measurement INstruments (COSMIN) checklist. The quality of the psychometric properties will be rated using the COSMIN checklist and qualitatively synthesised using the modified Grading of Recommendations Assessment, Development, and Evaluation checklist.

Ethics and dissemination: No ethical approvals are needed. The mixed review outputs will collectively inform the development, implementation, and evaluation of bespoke interventions for AYALHIV. Review outcomes will be disseminated in a peer-reviewed journal, on social media, and through policy briefs.

Registration: PROSPERO - CRD42022325172.

Keywords: Adolescents; HIV/AIDS; psychometrics; positive psychology; systematic review protocol

sub-Saharan Africa.

Strengths and limitations of this study:

The study strengths include:

- i) Application of a systematic methodology guided by standardised guidelines
- ii) Utilisation of multiple data sources
- iii) Article screening and data collection will be performed in duplicate and independently
- iv) Involvement of AYALHIV in the review and dissemination

For peer review only

- A weakness of the study is the exclusion of studies not published in English; this introduces language bias.

For peer review only

Introduction

HIV remains a global health problem, with low- and middle-income countries disproportionately affected.[1] Further, the burden of HIV in young people in low-resource settings, particularly in the sub-Saharan Africa (SSA) region, remains high.[2-4] Adolescence and early adulthood are challenging developmental stages, with the burden of navigating life challenges often even greater for adolescents and young adults living with HIV (AYALHIV, 10-24 years old).[1,2,5] For instance, AYALHIV face multiple biopsychosocial challenges, including stigma, negotiating reproductive health, socioeconomic deprivation, violence and other difficulties.[1,2,6] Also, with the increased availability and uptake of highly effective antiretroviral therapy, HIV has evolved into a long-term condition with a concomitant surge in comorbid non-communicable diseases.[5,7] For example, common mental disorders, including anxiety and depression, are prevalent in AYALHIV, with a pooled prevalence of 26.1% (95% CI 18.9-34.8).[2] However, there is a dearth of integrated programmes combining HIV and mental health care.[1,2,5] Importantly, many mental health conditions diagnosed in adults emerge in late adolescence and young adulthood, and effective management at this stage can prevent long-term mental illness.[2,5] Systematic reviews have demonstrated that access to mental healthcare by AYALHIV is associated with positive outcomes across the treatment continuum, including: increased treatment initiation, increased adherence to care, viral load control, reduced morbidity and mortality and retention in care.[1,5]

Mental health endpoints within HIV care have been traditionally conceptualised as improvements in negative psychiatric symptomatology.[6,8] For example, success in psychotherapies is invariably benchmarked against declines in anxiety, depression, post-traumatic stress disorders, and other negative psychological indices.[6] However, focusing on negative indices misses the opportunity to capture the multidimensionality of mental health. A holistic mental health evaluation requires a comprehensive focus on both negative and positive mental health constructs,[6,8] and recognition of this has resulted in a shift towards positive psychology, a framework that emphasises increasing human well-being and positive functioning.[6] Positive mental health interventions (PMHIs) are anchored upon the need to improve and evaluate human strengths and capabilities as enshrined in positive outcomes such as self-esteem, resilience, hope, self-worth, social resources, and flourishing.[6,8,9] For instance, studies have shown that people living with a chronic condition (e.g., HIV) develop resilience with time.[6,9] Resilience is defined as the "…*positive psychological, behavioural, and/or social adaptation in the face of stressors and adversities*…", and is a known buffer to stressful life events.[9] The resilience developed in navigating the challenges of living with a chronic condition is potentially transferable into everyday functioning.[9] Positive psychology interventions (e.g., resilience-building approaches) are central to prevention health promotion and act as an entry point to stepped-up care for mental problems in routine HIV care.[6]

With the proliferation of PMHIs comes the need to routinely evaluate the clinical endpoints from both the clients' and therapists' perspectives.[10] The patient-reported outcomes revolution is hinged upon assessing treatment success or lack thereof from the patient perspective.[8,10] Patient-reported outcomes facilitate patient-clinician communication and clinical decision-making; this is pivotal to patient-centred care.[8] The patient's evaluation of their health, treatment expectations and outcomes are contingent

For peer review only

upon the availability of validated and reliable outcome measures.[8] The last few decades have seen a proliferation of positive psychology outcome measures.[11] However, there is limited understanding of the salient positive psychological constructs linked to AYALHIV's improved well-being and health-related quality of life. Wayant et al. (2021), in their scoping review, mapped 15 positive psychological constructs associated with increased quality of life and survival in adolescents and young adults living with cancer.[12] Well-being, personal growth, hope, meaning in life, self-esteem, vitality and optimism were the most cited positive constructs;[12] these constructs are potentially transferable to AYALHIV. Conversely, etiological differences between cancer and HIV could also lead to differences in lived experiences, resulting in differential perceptions in positive psychological constructs.[12] For instance, HIV-related stigma (often associated with issues related to HIV's potential infectiousness) may have a greater impact on mental health functioning in AYALHIV.[13,14] when compared to the effects of cancer-related stigma.[15] It is thus critical to contextualise the impacts of positive psychological outcomes in AYALHIV. Govindasamy et al. (2021) performed a mixed-methods systematic review to explore correlates of well-being among AYALHIV in SSA to inform econometric evaluations.[13] The review showed that social support, belonging, purpose in life, and self-acceptance optimise well-being in AYALHIV.[13] Although the review provides essential insights, the sole focus on well-being limits our comprehensive understanding of the spectrum of positive psychological constructs in AYALHIV residing in SSA. There is a need to build on Govindasamy et al.'s work to understand positive psychological constructs in HIV care for AYALHIV aged 10-24 years, holistically. Also, there is a paucity of collective evidence of the psychometric robustness of the positive psychological outcomes used in AYALHIV. Some of the available generic outcomes may not comprehensively reflect the nuances of living with HIV.[13] This mixed review, therefore, seeks to:

1) identify positive psychological outcomes and corresponding outcome measures used in AYALHIV in SSA
2) critically appraise the psychometric properties of the identified positive psychology outcomes used in AYALHIV
3) map factors associated with the identified positive psychological constructs
4) glean an item bank from outcomes with robust psychometrics

The proposed review will map positive psychological constructs in AYALHIV in SSA by identifying commonly applied constructs and developing an evidence map/conceptual framework for measuring positive psychological outcomes. Further, the review will strengthen the measurement of positive mental health constructs to improve the well-being of AYALHIV. The review will also assist in identifying psychometrics that may require further evaluation and systematically identify factors influencing positive psychological function in AYALHIV, a group with disproportionately poor HIV treatment outcomes.[4,13]

More importantly, the initial item bank will inform the development of a context-specific positive psychology outcome measure for routine clinical and research use. The resultant outcome measure will contribute to the evidence base of the utility of positive psychological interventions in AYALHIV residing in low-income settings.

For peer review only

Methods

Overview

This mixed review will be done in two sequential and complementary phases. First, a scoping review will identify positive psychological outcomes used in AYALHIV in Sub-Saharan Africa and map the constructs onto the corresponding measures. The scoping review will be performed per Preferred Reporting Items for Systematic reviews and Meta-Analyses extension for Scoping Reviews (PRISMA-ScR) guidelines - See Additional File 1.[16] The second phase will systematically evaluate the psychometric properties of the outcomes identified from the scoping review. The evaluation of psychometrics of the outcome measures will be performed and reported according to the Preferred Reporting Items of Systematic Reviews and Meta-Analyses Protocol (PRISMA-P) guidelines - See Additional File 2.[17] Where appropriate, we will outline specific methodological considerations unique to each phase.

Protocol/ registration

The protocol was registered on the PROSPERO database - CRD42022325172.

Eligibility criteria

The following criteria will be applied in selecting articles:

Study designs/interventions

For the scoping review, we will include all quantitative designs, mixed methods, qualitative studies exploring the positive psychological phenomenon in AYALHIV in SSA, and grey literature (e.g., blogs, websites). For the systematic review, only quantitative designs will be included. Systematic reviews, editorials, case studies and study protocols will be excluded from the scoping and systematic reviews.

Participants/settings

For both phases of the mixed review, we will analyse all studies reporting on using and evaluating positive psychological constructs in AYALHIV (10-24 years old) in SSA across all settings. We focus on AYALHIV as it is the group with the greatest burden of HIV globally.[4,13] We anticipate that some studies will contain data on AYALHIV and other age bands (e.g., children, middle-aged adults). In such cases, an article will be considered for review if the average age is within the 10-24 years range or if over 50% of the participants are AYALHIV.

Language

We will restrict the analysis to articles published in English for both phases of the mixed review. We do not have the resources to analyse articles published in other languages.

Information sources

Peer-reviewed articles will be searched/retrieved from these electronic databases: PubMed, Scopus, Web of Science, Africa-Wide Information, CINAHL, PsychINFO, and Google Scholar. Databases will be searched from inception through September 30, 2022. Where only an abstract is available online, and information regarding psychometrics is neither clear nor available from the text, an attempt to contact the lead author will be made requesting the full article to ensure literature saturation and a

For peer review only

truthful rating. The article will be excluded from the review if there is no response in two weeks following three email reminders. We will also review grey literature using the Google Scholar search engine to search potential databases such as university databases, research reports, pre-prints, newsletters and bulletins, policy briefs, guidelines, and conference proceedings for articles. For completeness, we will also perform both backwards and forward searches of the reference lists of identified articles and databases, respectively. Finally, we will also contact experts implementing PMHIs to check for articles we may have missed using the proposed search strategy.

Search strategy

For the scoping review, as an illustration, articles in CINAHL will be searched using the AND Boolean logic operators, i.e., 1 AND 6 AND 9 AND 12 (Table 1). The search strategy will be amended for the systematic review component to include additional constructs identified through the scoping review.

<Insert Table 1 here>

Table 1: CINAL search strategy

Search #	Query
**1**	**adolescen* OR juvenile* OR teen* OR youth* OR “young person*” OR “young people” OR Adolescence OR Young Adult**
2	Hiv OR hiv-1* OR hiv-2*OR hiv1 OR hiv2 OR “HIV infect*” OR “human immunodeficiency virus” OR “human immunodeficiency virus” OR “human immuno-deficiency virus” OR “human immune-deficiency virus” OR “acquired immunodeficiency syndrome” OR “acquired immunodeficiency syndrome” OR “acquired immuno-deficiency syndrome” OR “acquired immune-deficiency syndrome” OR HIV Infections OR Human Immunodeficiency Virus
3	“human immun*” AND “deficiency virus”
4	hiv or aids or acquired human immunodeficiency syndrome or human immunodeficiency virus OR HIV/AIDS
5	“acquired immun*” AND “deficiency syndrome”
**6**	**2 OR 3 OR 4 0R 5**
7	hope* OR optimis* OR resilien* OR cope OR coping OR gratitude OR grateful OR happiness OR joy OR gladness OR satisf* OR “self efficacy” OR self-efficacy OR content*OR wellbeing OR well-being OR “self acceptance” OR self-acceptance OR “self esteem” OR self-esteem OR “self concept” OR self-concept OR “self confidence” OR self-confidence OR “self perception” OR self-perception OR “self worth” OR self-worth OR “personal growth” OR tranquil* OR perseverance OR vitality OR meaning OR “social* inclus*” OR “social participation” OR “social engagement” OR “social support” OR “self care” OR self-care OR “positive attitude” OR “positive thinking” OR “positive mindset” OR mindfulness OR empower*OR love OR spiritual* OR “community integration” OR “community participation” OR humour OR dignity OR pleasure OR creativ* OR transcend* OR goal* OR Psychological Well-Being OR Hope OR Optimism OR Coping OR Human Dignity OR Hardiness OR Adaptation, Psychological OR Happiness OR Personal Satisfaction OR Self Efficacy OR Self Concept OR Social Inclusion OR Social Participation OR Self Care OR Mindfulness OR Empowerment OR Love OR Spirituality OR Social Networks OR Pleasure OR Creativeness OR Self Transcendence OR (Goals and Objectives)

8	hope* OR optimis* OR optimism OR resilience OR resilien* OR resilient OR cope OR coping OR gratitude OR grateful OR happiness OR joy OR gladness OR (life satisfaction) OR satisfaction OR satisf* OR “self efficacy” OR self-efficacy OR content OR contentment OR content*OR wellbeing OR well-being OR “self acceptance” OR self-acceptance OR “self esteem” OR self-esteem OR “self concept” OR self-concept OR “self confidence” OR self-confidence OR “self perception” OR self-perception OR “self worth” OR self-worth OR “personal growth” OR tranquillity OR tranquil* OR perseverance OR vitality OR meaning OR “social* inclus*” OR “social participation” OR “social engagement” OR “social support” OR “self care” OR self-care OR “positive attitude” OR “positive thinking” OR “positive mindset” OR mindfulness OR empowerment OR empower*OR love OR spirituality OR spiritual* OR “community integration” OR “community participation” OR humour OR dignity OR pleasure OR creativ* OR transcend* OR goal* OR Psychological Well-Being OR Hope OR Optimism OR Coping OR Human Dignity OR Hardiness OR Adaptation, Psychological OR Happiness OR Personal Satisfaction OR Self Efficacy OR Self Concept OR Social Inclusion OR Social Participation OR Self Care OR Mindfulness OR Empowerment OR Love OR Spirituality OR Social Networks OR Pleasure OR Creativeness OR Self Transcendence OR (Goals and Objectives) OR growth mindset OR positive mindset OR (*mindset) OR flourishing OR flourish* OR thriving OR thriv*
**9**	**7 OR 8**
10	Angola or Benin or Botswana or Burkina Faso or Burundi or Cameroon or Cape Verde or Central African Republic or CHAD or Comoros or Congo or Congo Democratic Republic or Djibouti or Equatorial Guinea or Eritrea or Ethiopia or Gabon or Gambia or Ghana or Guinea or Guinea-Bissau or Cote d'Ivoire or Ivory Coast or Kenya or Lesotho or Liberia or Madagascar or Malawi or Mali or Mozambique or Namibia or Niger or Nigeria or Sao tome and Principe or Rwanda or Senegal or Seychelles or Sierra Leone or Somalia or South Africa or South Sudan or Sudan or Swaziland or Tanzania or Togo or Uganda or Zambia or Zimbabwe
11	‘Africa, South of the Sahara’ OR “sub-Saharan Africa”
**12**	**10 OR 11**

Adapted from[18]

Data management

Retrieved articles will be imported into the Mendeley reference manager, which is password-protected. The articles will also be synchronised onto Mendeley and Dropbox cloud storage platforms and backed-up onto a password-encrypted external hard drive. All collaborators will have full access/administrative privileges to the shared Dropbox folder for the present systematic review. A trail/history of the electronic searches will also be saved on users' PubMed, Scopus, and EBSCOhost accounts. We will also print summaries of all the searches to enhance the data capturing of the search records.

Data collection process

The data collection process will be conducted in three stages, i.e., article retrieval, screening, and data extraction. These processes will invariably be similar for the scoping and systematic review phases. Here we describe these processes and highlight, where appropriate, differences in the two phases of the review.

Article retrieving

Two researchers (SS & VS) will independently search articles using a pre-defined search strategy. The lead author (JMD) will then import the searches into Mendeley and remove duplicates.

Screening

Upon completion of article retrieving, another set of independent researchers (SB & WM) will screen the articles by title and abstract using Rayyan software.[19] To increase methodological rigour, both researchers will independently review all retrieved articles, including documenting reasons for exclusion. Rayyan software automatically collates the number of hits assigned different ratings by the reviewers. Discrepancies will be resolved through discussion, and where consensus is not reached, a

For peer review only

more senior researcher (WM) will make the final decision. JMD and SS will then perform backwards and forward citation searches to identify other potential articles. Two senior researchers (FMC & WM) will review the list of identified articles afterwards to check for the completeness of the searches.

Data extraction

Once searches are finalised, two researchers (FM and NW) will retrieve the full articles and independently extract data from articles meeting the inclusion criteria. Data extraction will be performed in duplicate. Disagreements during data extraction will be resolved through consensus, and more senior researchers (FMC & WM) will make the final decisions if any impasses occur. For both phases of the review, we will extract the following information per study: research setting and design, study sample and participants' demographics. For the scoping review, we will additionally extract information on the conceptualisations/definitions and factors associated with positive psychological constructs. For the systematic review component, we will extract information on the mode of administration, the number of items, descriptions of domains, scoring and interpretation of scores and whether measures are free to use or require a license fee or other payment.

Charting/Outcomes and prioritisation

The conceptualisation of positive psychological constructs and the psychometric properties of the identified outcomes will be the primary outcome measures for the scoping and systematic review phases, respectively. For the systematic review, the clinical utility of the identified outcome measures will be the secondary outcome. See Table 2 for operational definitions of psychometric properties for the systematic review component.[18,20]

< Insert Table 2 here>

Table 2: Operational definitions of psychometric properties[18,20]

Term			Definition
**Domain**	**Measurement property**	**Aspect of a measurement property**	
**Reliability**			The degree to which the measurement is free from measurement error
**Reliability (extended definition)**			The extent to which scores for patients who have not changed are the same for repeated measurement under several conditions: e.g., using different sets of items from the same PROM (internal consistency); over time (test-retest); by different persons on the same occasion (inter-rater); or by the same persons (i.e., raters or responders) on different occasions (intra-rater)
	**Internal consistency**		The degree of the interrelatedness among the items
	**Reliability**		The proportion of the total variance in the measurements which is due to 'true'† differences between patients
	**Measurement error**		The systematic and random error of a patient's score that is not attributed to true changes in the construct to be measured

**Validity**			The degree to which a PROM measures the construct(s) it purports to measure
	**Content validity**		The degree to which the content of a PROM is an adequate reflection of the construct to be measured
		**Face validity**	The degree to which (the items of) a PROM indeed looks as though they are an adequate reflection of the construct to be measured
	**Construct validity**		The degree to which the scores of a PROM are consistent with hypotheses *(for instance with regard to internal relationships, relationships to scores of other instruments, or differences between relevant groups)* based on the assumption that the PROM validly measures the construct to be measured
		**Structural validity**	The degree to which the scores of a PROM are an adequate reflection of the dimensionality of the construct to be measured
		**Hypotheses testing**	Item construct validity
		**Cross-cultural validity**	The degree to which the performance of the items on a translated or culturally adapted PROM are an adequate reflection of the performance of the items of the original version of the PROM
	**Criterion validity**		The degree to which the scores of a PROM are an adequate reflection of a 'gold standard'
**Responsiveness**			The ability of a PROM to detect change over time in the construct to be measured
	**Responsiveness**		Item responsiveness
**Interpretability***			Interpretability is the degree to which one can assign qualitative meaning - that is, clinical or commonly understood connotations – to a PROM's quantitative scores or change in scores.

† The word 'true' must be seen in the context of the CTT, which states that any observation is composed of two components – a true score and error associated with the observation. 'True' is the average score that would be obtained if the scale were given an infinite number of times. It refers only to the consistency of the score, and not to its accuracy.

** Interpretability is not considered a measurement property, but an important characteristic of a measurement instrument*

Risk of bias - individual studies

The scoping review aims to understand the conceptualisation of AYALHIV's positive psychological constructs. Consequently, we will not perform any risk of bias (RoB) assessments. However, the systematic review component aims to synthesise the evidence of psychometric robustness necessitating RoB assessment. We will use the revised COnsensus-based Standards for the selection of health Measurement INstruments (COSMIN) checklist to assess the RoB across studies retrieved for psychometric evaluation.[18,20] The COSMIN methodology consists of three steps. The checklist consists of methodological benchmarks for ten (10) psychometric properties, which are categorised into three major groups, i.e. content validity (e.g. patient-reported outcome measure development), internal structure (e.g. structural validity), and other psychometrical properties (e.g. criterion validity).[18,20] Each psychometric property is rated using a pre-set criterion, and using the principle of "worse score counts", the lowest rating is ascribed as the overall methodological quality rating.[18] Methodological quality is

For peer review only

rated on a four-point Likert scale, i.e. "inadequate", "doubtful", "adequate", and "very good"; the higher the rating, the lower the risk of bias.[18,20] We anticipate that not all details may be recorded for the retrieved articles, especially for studies whose primary aim was not psychometric evaluation. We will, therefore, contact the corresponding author to achieve the most truthful rating of the psychometric property to decrease bias during analysis.

Quality of psychometric properties and data extraction

The quality of psychometrical properties will be evaluated using an updated, hybrid checklist based on previous work by Terwee et al.[21] and Prinsen et al.[22] (See Table 3). Each psychometric property will be rated as; sufficient (+), insufficient (–), or indeterminate (?).[20] Positive ratings represent high-quality psychometrics.[20]

< Insert Table 3 here>

Table 3: Updated criteria for good measurement properties [21,22]

Measurement property	Rating	Criteria
Structural validity	+	**CTT:**<br>CFA: CFI or TLI or comparable measure >0.95 OR RMSEA <0.06 OR SRMR <0.08<br>**IRT/Rasch**:<br>No violation of unidimensionality: CFI or TLI or comparable measure >0.95 OR RMSEA <0.06 OR SRMR <0.08<br>*AND*<br>no violation of local independence: residual correlations among the items after controlling for the dominant factor < 0.20 OR Q3's < 0.37<br>*AND*<br>no violation of monotonicity: adequate looking graphs OR item scalability >0.30<br>*AND*<br>adequate model fit:<br>IRT: χ^2 >0.01<br>Rasch: infit and outfit mean squares ≥ 0.5 and ≤ 1.5 OR Z-standardised values > -2 and <2
	?	CTT: Not all information for '+' reported<br>IRT/Rasch: Model fit not reported
	–	Criteria for '+' not met
Internal consistency	+	At least low evidence for sufficient structural validity AND Cronbach's alpha(s) ≥ 0.70 for each unidimensional scale or subscale[6]
	?	Criteria for "At least low evidence for sufficient structural validity" not met
	–	At least low evidence for sufficient structural validity AND Cronbach's alpha(s) < 0.70 for each unidimensional scale or subscale
Reliability	+	ICC or weighted Kappa ≥ 0.70
	?	ICC or weighted Kappa not reported
	–	ICC or weighted Kappa < 0.70

For peer review only

Measurement error	+	SDC or LoA < MIC
	?	MIC not defined SDC or LoA
	–	> MIC
Hypotheses testing for construct validity	+	The result is in accordance with the hypothesis
	?	No hypothesis defined (by the review team)
	–	The result is not in accordance with the hypothesis
Cross-cultural validity\measurement invariance	+	No important differences found between group factors (such as age, gender, language) in multiple group factor analysis OR no important DIF for group factors (McFadden's R^2 < 0.02)
	?	No multiple group factor analysis OR DIF analysis performed
	–	Important differences between group factors OR DIF was found
Criterion validity	+	Correlation with gold standard ≥ 0.70 OR AUC ≥ 0.70
	?	Not all information for '+' reported
	–	Correlation with gold standard < 0.70 OR AUC < 0.70
Responsiveness	+	The result is in accordance with the hypothesis OR AUC ≥ 0.70
	?	No hypothesis defined (by the review team)
	–	The result is not in accordance with the hypothesis OR AUC < 0.70

Key: AUC = area under the curve, CFA = confirmatory factor analysis, CFI = comparative fit index, CTT = classical test theory, DIF = differential item functioning, ICC = intraclass correlation coefficient, IRT = item response theory, LoA = limits of agreement, MIC = minimal important change, RMSEA: Root Mean Square Error of Approximation, SEM = Standard Error of Measurement, SDC= smallest detectable change, SRMR: Standardised Root Mean Residuals, TLI = Tucker-Lewis index.

Best evidence synthesis

Initially, we will develop a conceptual framework for synthesising both qualitative and quantitative studies retrieved. We will then apply a narrative synthesis to map the key "themes/constructs" emerging from the scoping review. The conceptual framework/model will map the constructs and subsequently guide the psychometric evaluation. The collective evidence per psychometric property per outcome will be synthesised using the modified Grading of Recommendations Assessment, Development, and Evaluation (GRADE) checklist,[23] as outlined in Table 4. The modified GRADE will then be used to collate the RoB results and the quality of psychometric ratings to qualitatively synthesise/summarise the quality of evidence per psychometric property across studies. The quality of evidence per psychometrical property will be classified as very low, low, moderate or high.[23]

<Insert Table 4 here>

Table 4: GRADE checklist- best evidence synthesis[23]

Quality level	Definition/criterion
High	We are very confident that the true measurement property lies close to that of the estimate of the measurement property
Moderate	We are moderately confident in the measurement property estimate: the true measurement property is likely to be close to the estimate of the measurement property, but there is a possibility that it is substantially different

For peer review only

For peer review only - http://bmjopen.bmj.com/site/about/guidelines.xhtml

Low	Our confidence in the measurement property estimate is limited: the true measurement property may be substantially different from the estimate of the measurement property
Very low	We have very little confidence in the measurement property estimate: the true measurement property is likely to be substantially different from the estimate of the measurement property

Patient and Public Involvement statement

We will work collaboratively with AYALHIV during data collection and dissemination. AYALHIV representatives previously trained and involved in systematic reviews will assist with article screening. We will co-create the dissemination plans; for instance, adolescents and young adults with lived experiences will be involved in co-developing output animation and contributing to the project blogs, amongst other dissemination activities.

Ethics and dissemination

No ethical approvals are needed as this is a secondary study. The proposed mixed review will map and appraise the collective evidence of the psychometric robustness of positive psychological outcomes used in AYALHIV. The proposed review builds on recommendations of systematic reviews on the need to measure positive psychological constructs across diverse populations objectively. This is important given the need to use valid and reliable outcomes in understanding the positive effects of living with HIV. The review will also assist in identifying psychometrically robust outcomes to inform an item bank to adapt a context-specific outcome measure for AYALHIV in low-resource settings. For example, we will consolidate all self-esteem outcome measures and categorise items from multiple outcomes into common factors/"themes". Also, the review will systematically identify factors influencing well-being in AYALHIV. The outputs will collectively inform the development, implementation, and evaluation of bespoke positive mental health interventions for AYALHIV, hence a need for a multimodal dissemination plan to reach multiple stakeholders. This review is attached to ongoing work in which AYALHIV are collaboratively engaged. It is part of a larger study to explore various constructs to understand how they improve AYALHIV's health outcomes. We have already recruited AYALHIV to serve as a Youth Expert Panel (YEP). The YEP functions as both a guide to the study/research process and an additional group of analysts and discussants to examine the emerging analysis and findings. We will publish the outcomes in a peer-reviewed journal. Additionally, we will disseminate the outcomes through social media, policy briefs and blogs.

Abbreviations

AYALHIV- adolescents and young adults living with HIV; CINAHL- Cumulative Index of Nursing and Allied Health Literature; COSMIN - COnsensus-based Standards for the selection of health Measurement Instruments; GRADE- Grading of Recommendations Assessment, Development, and Evaluation; HRQoL - health-related quality of life; PRISMA-P - Preferred Reporting Items of Systematic Reviews and Meta-Analyses Protocol.

For peer review only

Declarations

Ethics approval and consent to participate
Not applicable.

Consent for publication
Not applicable.

Availability of data and materials
Not applicable for a review protocol. However, all relevant data/materials, including data collection tools, will be submitted as supplementary files during the publication of the full review.

Competing interests
All the authors declare no competing interests.

Funding
This research was funded in whole or in part by Templeton World Charity Foundation, Inc. [TWCF-2021-20629]. For the purpose of open access, the author has applied a CC-BY public copyright license to any author accepted manuscript version arising from this submission. The funder had no role in study design, data collection, data analysis, data interpretation, or writing of the report.

Authors' contributions
JMD was primarily responsible for protocol writing. JMD, FMC, FM, SS, VC, NW, SB, and WM were involved in the conceptualisation of the study and editing all protocol manuscript versions. JMD will search the literature and data management. FMC, FM, SS, VC, NW, SB, and WM will be responsible for article screening and quality assurance, data extraction and qualitative synthesis.

Acknowledgements
We would like to acknowledge Alison Derbyshire, an Academic Liaison & Training Specialist at the Liverpool School of Tropical Medicine Library, for assisting with refining the search strategies.

Author's ORCID information
- Jermaine M. Dambi: https://orcid.org/0000-0002-2446-7903
- Frances M. Cowan: https://orcid.org/0000-0003-3087-4422
- Faith Martin: https://orcid.org/0000-0002-0141-1210
- Sharon Sibanda: https://orcid.org/0000-0002-6595-4097
- Victoria Simms: https://orcid.org/0000-0002-4897-458X
- Nicola Willis: https://orcid.org/0000-0003-0452-0196
- Sarah Bernays: https://orcid.org/0000-0001-7628-8408
- Webster Mavhu: https://orcid.org/0000-0003-1881-4398

References

1. Bhana A, Abas MA, Kelly J, van Pinxteren M, Mudekunye LA, Pantelic M. Mental health interventions for adolescents living with HIV or affected by HIV in low- and middle-income countries: systematic review. BJPsych Open. Sep 4 2020;6(5):e104. doi:10.1192/bjo.2020.67

2. Ayano G, Demelash S, Abraha M, Tsegay L. The prevalence of depression among adolescent with HIV/AIDS: a systematic review and meta-analysis. AIDS Res Ther. Apr 27 2021;18(1):23. doi:10.1186/s12981-021-00351-1

For peer review only

3. Dow DE, Mmbaga BT, Turner EL, et al. Building resilience: a mental health intervention for Tanzanian youth living with HIV. AIDS Care. 2018;30(sup4):12-20. doi:10.1080/09540121.2018.1527008

4. Mavhu W, Willis N, Mufuka J, et al. Effect of a differentiated service delivery model on virological failure in adolescents with HIV in Zimbabwe (Zvandiri): a cluster-randomised controlled trial. Lancet Glob Health. Feb 2020;8(2):e264-e275. doi:10.1016/S2214-109X(19)30526-1

5. Bhana A, Kreniske P, Pather A, Abas MA, Mellins CA. Interventions to address the mental health of adolescents and young adults living with or affected by HIV: state of the evidence. J Int AIDS Soc. Jun 2021;24 Suppl 2:e25713. doi:10.1002/jia2.25713

6. Bolier L, Haverman M, Westerhof GJ, Riper H, Smit F, Bohlmeijer E. Positive psychology interventions: a meta-analysis of randomised controlled studies. BMC Public Health. Feb 8 2013;13:119. doi:10.1186/1471-2458-13-119

7. Adeyemi O, Lyons M, Njim T, et al. Integration of non-communicable disease and HIV/AIDS management: a review of healthcare policies and plans in East Africa. BMJ Glob Health. May 2021;6(5)doi:10.1136/bmjgh-2020-004669

8. Bristowe K, Murtagh FEM, Clift P, et al. The development and cognitive testing of the positive outcomes HIV PROM: a brief novel patient-reported outcome measure for adults living with HIV. Health Qual Life Outcomes. Jul 6 2020;18(1):214. doi:10.1186/s12955-020-01462-5

9. Dulin AJ, Dale SK, Earnshaw VA, et al. Resilience and HIV: a review of the definition and study of resilience. AIDS Care. Aug 2018;30(sup5):S6-S17. doi:10.1080/09540121.2018.1515470

10. Kall M, Marcellin F, Harding R, Lazarus JV, Carrieri P. Patient-reported outcomes to enhance person-centred HIV care. Lancet HIV. Jan 2020;7(1):e59-e68. doi:10.1016/S2352-3018(19)30345-5

11. Stoner CR, Stansfeld J, Orrell M, Spector A. The development of positive psychology outcome measures and their uses in dementia research: A systematic review. Dementia (London). Aug 2019;18(6):2085-2106. doi:10.1177/1471301217740288

12. Wayant C, Manquen J, Wendelbo H, et al. Identification of Evidence for Key Positive Psychological Constructs in Pediatric and Adolescent/Young Adult Patients with Cancer: A Scoping Review. J Adolesc Young Adult Oncol. Jun 2021;10(3):247-259. doi:10.1089/jayao.2020.0184

13. Govindasamy D, Seeley J, Olaru ID, Wiyeh A, Mathews C, Ferrari G. Informing the measurement of wellbeing among young people living with HIV in sub-Saharan Africa for policy evaluations: a mixed-methods systematic review. Health Qual Life Outcomes. May 5 2020;18(1):120. doi:10.1186/s12955-020-01352-w

14. Rich C, Mavhu W, France NF, et al. Exploring the beliefs, experiences and impacts of HIV-related self-stigma amongst adolescents and young adults living with HIV in Harare, Zimbabwe: A qualitative study. PLoS One. 2022;17(5):e0268498. doi:10.1371/journal.pone.0268498

15. Munn Z, Stern C, Aromataris E, Lockwood C, Jordan Z. What kind of systematic review should I conduct? A proposed typology and guidance for systematic reviewers in the medical and health sciences. BMC Med Res Methodol. Jan 10 2018;18(1):5. doi:10.1186/s12874-017-0468-4

16. Tricco AC, Lillie E, Zarin W, et al. PRISMA Extension for Scoping Reviews (PRISMA-ScR): Checklist and Explanation. Ann Intern Med. Oct 2 2018;169(7):467-473. doi:10.7326/M18-0850

17. Page MJ, McKenzie JE, Bossuyt PM, et al. The PRISMA 2020 statement: an updated guideline for reporting systematic reviews. BMJ. Mar 29 2021;372:n71. doi:10.1136/bmj.n71

For peer review only

18. Terwee CB, Prinsen CAC, Chiarotto A, et al. COSMIN methodology for evaluating the content validity of patient-reported outcome measures: a Delphi study. Qual Life Res. May 2018;27(5):1159-1170. doi:10.1007/s11136-018-1829-0

19. Ouzzani M, Hammady H, Fedorowicz Z, Elmagarmid A. Rayyan-a web and mobile app for systematic reviews. Syst Rev. Dec 5 2016;5(1):210. doi:10.1186/s13643-016-0384-4

20. Mokkink LB, de Vet HCW, Prinsen CAC, et al. COSMIN Risk of Bias checklist for systematic reviews of Patient-Reported Outcome Measures. Qual Life Res. May 2018;27(5):1171-1179. doi:10.1007/s11136-017-1765-4

21. Terwee CB, Bot SD, de Boer MR, et al. Quality criteria were proposed for measurement properties of health status questionnaires. J Clin Epidemiol. Jan 2007;60(1):34-42. doi:10.1016/j.jclinepi.2006.03.012

22. Prinsen CA, Vohra S, Rose MR, et al. How to select outcome measurement instruments for outcomes included in a "Core Outcome Set" - a practical guideline. Trials. Sep 13 2016;17(1):449. doi:10.1186/s13063-016-1555-2

23. Zhang Y, Coello PA, Guyatt GH, et al. GRADE guidelines: 20. Assessing the certainty of evidence in the importance of outcomes or values and preferences-inconsistency, imprecision, and other domains. J Clin Epidemiol. Jul 2019;111:83-93. doi:10.1016/j.jclinepi.2018.05.011

For peer review only

Preferred Reporting Items for Systematic reviews and Meta-Analyses extension for Scoping Reviews (PRISMA-ScR) Checklist

SECTION	ITEM	PRISMA-ScR CHECKLIST ITEM	REPORTED ON PAGE #
**TITLE**			
Title	1	Identify the report as a scoping review.	1
**ABSTRACT**			
Structured summary	2	Provide a structured summary that includes (as applicable): background, objectives, eligibility criteria, sources of evidence, charting methods, results, and conclusions that relate to the review questions and objectives.	2
**INTRODUCTION**			
Rationale	3	Describe the rationale for the review in the context of what is already known. Explain why the review questions/objectives lend themselves to a scoping review approach.	4-5
Objectives	4	Provide an explicit statement of the questions and objectives being addressed with reference to their key elements (e.g., population or participants, concepts, and context) or other relevant key elements used to conceptualize the review questions and/or objectives.	4
**METHODS**			
Protocol and registration	5	Indicate whether a review protocol exists; state if and where it can be accessed (e.g., a Web address); and if available, provide registration information, including the registration number.	6
Eligibility criteria	6	Specify characteristics of the sources of evidence used as eligibility criteria (e.g., years considered, language, and publication status), and provide a rationale.	6
Information sources*	7	Describe all information sources in the search (e.g., databases with dates of coverage and contact with authors to identify additional sources), as well as the date the most recent search was executed.	6-7
Search	8	Present the full electronic search strategy for at least 1 database, including any limits used, such that it could be repeated.	7-8
Selection of sources of evidence†	9	State the process for selecting sources of evidence (i.e., screening and eligibility) included in the scoping review.	6-7
Data charting process‡	10	Describe the methods of charting data from the included sources of evidence (e.g., calibrated forms or forms that have been tested by the team before their use, and whether data charting was done independently or in duplicate) and any processes for obtaining and confirming data from investigators.	9
Data items	11	List and define all variables for which data were sought and any assumptions and simplifications made.	9
Critical appraisal of individual sources of evidence§	12	If done, provide a rationale for conducting a critical appraisal of included sources of evidence; describe the methods used and how this information was used in any data synthesis (if appropriate).	12

For peer review only - http://bmjopen.bmj.com/site/about/guidelines.xhtml

SECTION	ITEM	PRISMA-ScR CHECKLIST ITEM	REPORTED ON PAGE #
Synthesis of results	13	Describe the methods of handling and summarizing the data that were charted.	12
**RESULTS**			
Selection of sources of evidence	14	Give numbers of sources of evidence screened, assessed for eligibility, and included in the review, with reasons for exclusions at each stage, ideally using a flow diagram.	n/a
Characteristics of sources of evidence	15	For each source of evidence, present characteristics for which data were charted and provide the citations.	N/A
Critical appraisal within sources of evidence	16	If done, present data on critical appraisal of included sources of evidence (see item 12).	N/A
Results of individual sources of evidence	17	For each included source of evidence, present the relevant data that were charted that relate to the review questions and objectives.	N/A
Synthesis of results	18	Summarize and/or present the charting results as they relate to the review questions and objectives.	N/A
**DISCUSSION**			
Summary of evidence	19	Summarize the main results (including an overview of concepts, themes, and types of evidence available), link to the review questions and objectives, and consider the relevance to key groups.	9
Limitations	20	Discuss the limitations of the scoping review process.	N/A
Conclusions	21	Provide a general interpretation of the results with respect to the review questions and objectives, as well as potential implications and/or next steps.	N/A
**FUNDING**			
Funding	22	Describe sources of funding for the included sources of evidence, as well as sources of funding for the scoping review. Describe the role of the funders of the scoping review.	9

JBI = Joanna Briggs Institute; PRISMA-ScR = Preferred Reporting Items for Systematic reviews and Meta-Analyses extension for Scoping Reviews.

* Where *sources of evidence* (see second footnote) are compiled from, such as bibliographic databases, social media platforms, and Web sites.

† A more inclusive/heterogeneous term used to account for the different types of evidence or data sources (e.g., quantitative and/or qualitative research, expert opinion, and policy documents) that may be eligible in a scoping review as opposed to only studies. This is not to be confused with *information sources* (see first footnote).

‡ The frameworks by Arksey and O'Malley (6) and Levac and colleagues (7) and the JBI guidance (4, 5) refer to the process of data extraction in a scoping review as data charting.

§ The process of systematically examining research evidence to assess its validity, results, and relevance before using it to inform a decision. This term is used for items 12 and 19 instead of "risk of bias" (which is more applicable to systematic reviews of interventions) to include and acknowledge the various sources of evidence that may be used in a scoping review (e.g., quantitative and/or qualitative research, expert opinion, and policy document).

From: Tricco AC, Lillie E, Zarin W, O'Brien KK, Colquhoun H, Levac D, et al. PRISMA Extension for Scoping Reviews (PRISMAScR): Checklist and Explanation. Ann Intern Med. 2018;169:467–473. doi: 10.7326/M18-0850.

For peer review only

PRISMA-P 2015 Checklist

This checklist has been adapted for use with protocol submissions to *Systematic Reviews* from Table 3 in Moher D et al: Preferred reporting items for systematic review and meta-analysis protocols (PRISMA-P) 2015 statement. *Systematic Reviews* 2015 **4**:1

Section/topic	#	Checklist item	Information reported		Line number(s)
			Yes	No	
**ADMINISTRATIVE INFORMATION**					
**Title**					
Identification	1a	Identify the report as a protocol of a systematic review	☒	☐	1
Update	1b	If the protocol is for an update of a previous systematic review, identify as such	☐	☒	N/A
**Registration**	2	If registered, provide the name of the registry (e.g., PROSPERO) and registration number in the Abstract	☒	☐	6
**Authors**					
Contact	3a	Provide name, institutional affiliation, and e-mail address of all protocol authors; provide physical mailing address of corresponding author	☒	☐	1
Contributions	3b	Describe contributions of protocol authors and identify the guarantor of the review	☒	☐	14
**Amendments**	4	If the protocol represents an amendment of a previously completed or published protocol, identify as such and list changes; otherwise, state plan for documenting important protocol amendments	☐	☒	N/A
**Support**					
Sources	5a	Indicate sources of financial or other support for the review	☒	☐	14
Sponsor	5b	Provide name for the review funder and/or sponsor	☒	☐	14
Role of sponsor/funder	5c	Describe roles of funder(s), sponsor(s), and/or institution(s), if any, in developing the protocol	☒	☐	14
**INTRODUCTION**					
**Rationale**	6	Describe the rationale for the review in the context of what is already known	☒	☐	4-5
**Objectives**	7	Provide an explicit statement of the question(s) the review will address with reference to participants, interventions, comparators, and outcomes (PICO)	☒	☐	5

For peer review only

For peer review only - http://bmjopen.bmj.com/site/about/guidelines.xhtml

Section/topic	#	Checklist item	Information reported		Line number(s)
			Yes	No	
**METHODS**					
**Eligibility criteria**	8	Specify the study characteristics (e.g., PICO, study design, setting, time frame) and report characteristics (e.g., years considered, language, publication status) to be used as criteria for eligibility for the review	☒	☐	6
**Information sources**	9	Describe all intended information sources (e.g., electronic databases, contact with study authors, trial registers, or other grey literature sources) with planned dates of coverage	☒	☐	6-7
**Search strategy**	10	Present draft of search strategy to be used for at least one electronic database, including planned limits, such that it could be repeated	☒	☐	7-8
STUDY RECORDS					
Data management	11a	Describe the mechanism(s) that will be used to manage records and data throughout the review	☒	☐	8
Selection process	11b	State the process that will be used for selecting studies (e.g., two independent reviewers) through each phase of the review (i.e., screening, eligibility, and inclusion in meta-analysis)	☒	☐	8
Data collection process	11c	Describe planned method of extracting data from reports (e.g., piloting forms, done independently, in duplicate), any processes for obtaining and confirming data from investigators	☒	☐	9
**Data items**	12	List and define all variables for which data will be sought (e.g., PICO items, funding sources), any pre-planned data assumptions and simplifications	☒	☐	9-10
**Outcomes and prioritization**	13	List and define all outcomes for which data will be sought, including prioritization of main and additional outcomes, with rationale	☒	☐	9
**Risk of bias in individual studies**	14	Describe anticipated methods for assessing risk of bias of individual studies, including whether this will be done at the outcome or study level, or both; state how this information will be used in data synthesis	☒	☐	10-11
DATA					
**Synthesis**	15a	Describe criteria under which study data will be quantitatively synthesized	☐	☒	N/A
	15b	If data are appropriate for quantitative synthesis, describe planned summary measures, methods of handling data, and methods of combining data from studies, including any planned exploration of consistency (e.g., I^2, Kendall's tau)	☐	☒	N/A
	15c	Describe any proposed additional analyses (e.g., sensitivity or subgroup analyses, meta-regression)	☐	☒	N/A
	15d	If quantitative synthesis is not appropriate, describe the type of summary planned	☒	☐	11-12

BioMed Central
The Open Access Publisher

Section/topic	#	Checklist item	Information reported		Line number(s)
			Yes	No	
**Meta-bias(es)**	16	Specify any planned assessment of meta-bias(es) (e.g., publication bias across studies, selective reporting within studies)	☐	☒	N/A
**Confidence in cumulative evidence**	17	Describe how the strength of the body of evidence will be assessed (e.g., GRADE)	☒	☐	12

For peer review only

BioMed Central
The Open Access Publisher
